# Awareness Level of Hypoglycemia Among Diabetes Mellitus Type 2 Patients in Al Qassim Region

**DOI:** 10.7759/cureus.35285

**Published:** 2023-02-22

**Authors:** Adel AlTowayan, Seetah Alharbi, Maryam Aldehami, Rand Albahli, Sama Alnafessah, Abeer M Alharbi

**Affiliations:** 1 Endocrinology and Diabetes, King Fahad Specialist Hospital, Buraidah, SAU; 2 Internal Medicine, King Fahad Specialist Hospital, Buraidah, SAU; 3 Family and Community Medicine, Qassim University, Qassim, SAU

**Keywords:** saudi arabia, qassim region, diabetes mellitus, hypoglycemia, knowledge of hypoglycemia

## Abstract

Background

Hypoglycemia has a major impact on patient health and glycemic management during insulin therapy for both type 1 (T1DM) and type 2 diabetes mellitus (T2DM). It is the rate-limiting complication in diabetes management that prevents stringent glucose control.

Objectives

To assess the knowledge and awareness about hypoglycemia as a complication of T2D in adults in Al Qassim, Saudi Arabia.

Methods

This is a cross-sectional study done among type 2 diabetes patients in Al-Qassim, Kingdom of Saudi Arabia, from January to June 2022. A previously validated online questionnaire was disseminated through social media to gather information from respondents. Participants were chosen via a simple random sampling technique. Data analysis was completed using SPSS (version 23; IBM Corp., Armonk, NY).

Results

Overall, 213 respondents were included in our study. The majority of them were females (70.9%). The participants' average age was 35.9 + 13.0 years. Our results revealed that the average awareness score of the study population was found to be 3.6 ± 1.1 (by using the Clarke method) and 3.7 ± 2.1 (by using the Gold method). Moreover, we found that impaired awareness of hypoglycemia’s prevalence by Clarke's questionnaire was 52.1% and 53.5% by using the Gold questionnaire. In addition, almost half of the respondents reported weakness as a symptom of hypoglycemia over the last six months and unconsciousness over the last 12 months. Hypertension was the most commonly reported chronic disease by our participants. Lastly, factors such as age, gender, educational level, geographic distribution, and history of chronic illness did not show any significant association with impaired awareness of the prevalence of hypoglycemia.

Conclusion

According to our research, we concluded that patients with type 2 diabetes mellitus in the region of Al-Qassim, Saudi Arabia, had insufficient knowledge about hypoglycemia as a complication of T2D. Moreover, the impaired awareness of hypoglycemia in diabetic patients was found to be high. Hence, there is a need for interventional programs to raise public awareness.

## Introduction

Diabetes mellitus is a chronic disease that affects the body's metabolism and is defined by hyperglycemia caused by the impairment of insulin secretion, increased peripheral resistance, or both. Diabetes is caused by an imbalance in carbohydrate, protein, and lipid metabolism as a result of complete or partial insulin secretion and/or activity. Type I diabetes, which is insulin-dependent, and type II diabetes, which is insulin-independent, are the two main types of diabetes [1,2]. This disease has become an epidemic all around the world. As of 2019, DM had affected 463 million people around the world and more, and it was expected to rise to 700 million by 2045 [3]. According to an epidemiological study of Saudis aged 15 and up from various areas of KSA, the age-adjusted prevalence (using WHO criteria) was higher in urban areas (males 12%, females 14%) than in rural areas (7% male and 7.7% female). The frequency was 29% among rural females of similar ages. Throughout the course of the study, 56% of those diagnosed with diabetes had no prior knowledge of their condition. In another study, it was discovered that 17% of people aged 30 and up had DM [4].

In most developed countries, diabetes is the fourth-leading cause of mortality. Diabetes complications like coronary artery disease and vascular disease, acute ischemic stroke, peripheral neuropathy leading to chronic wounds and amputations, acute kidney injury, and loss of vision are causing increased disability, lower life quality, and massive medical expenses in almost every culture. It is undoubtedly one of the most difficult health problems of the twenty-first century. On the other hand, the acute consequences of diabetes include diabetic ketoacidosis (DKA), hyperosmolar hyperglycemic nonketotic coma (HHNC), and hypoglycemia. DKA and HHNC are associated with insulin deficiency [5]. In hypoglycemia, where blood glucose ≤70 mg/dL, this is a serious diabetic complication with high individual and community costs [6]. Furthermore, Insulin-induced hypoglycemia is one of the typical side effects of insulin treatment. According to the data, insulin-related hypoglycemia results in about 98 thousand emergency room visits annually, as well as 30.000 hospitalizations in the US [7]. Preventing or limiting its occurrence and impact necessitates a thorough understanding of the implications [6]. Despite the fact that hypoglycemia occurs more commonly in people with type 1 diabetes, hypoglycemia requiring emergency medical intervention is just as common in people with type 2 diabetes as in people with type 1 diabetes, according to previous research [8]. Hypoglycemia causes neurogenic symptoms including tremors, palpitations, anxiety/arousal, sweating, hunger, and paresthesias. In addition to neuroglycopenic symptoms, including dizziness, weakness, drowsiness, delirium, confusion, and, at lower plasma glucose concentrations, seizure, and coma [9,10]. There are many long- and short-term complications of hypoglycemia in diabetic patients, and it can affect many systems in the body, including cerebrovascular, cardiovascular, retinal cell death, vision loss, and neurocognitive impairment, as well as health-related quality-of-life difficulties [11]. Furthermore, older diabetes patients who had hypoglycemia had a two-fold greater risk of falling [12]. As a result, there is a six-fold higher chance of dying from diabetes in patients with severe hypoglycemia than in those who do not have it [11].

Hypoglycemia has an impact on medical resources, leading to consumption and productivity losses that are significantly costly, and if this cost burden is not taken into consideration, diabetes care might be underestimated [13]. According to previous observational studies, the hypoglycemia risk is greater in insulin-treated patients who have been diabetic for a long time and have been on insulin for a long period of time [10,14,15]. Furthermore, severe hypoglycemia has been shown to happen up to five times more often in individuals with poor awareness [16]. Insufficient food consumption (47%), physical exercise (23%), insulin dose miscalculation (16%), and impaired hypoglycemia awareness (5%) were the most common causes of severe hypoglycemia, according to the findings of the Kedia N study [17]. Moreover, according to prospective research evaluating the hypoglycemic frequency and associated symptoms, participants with reduced awareness suffered more moderate and severe hypoglycemic episodes [18]. Research was conducted in South India, and 366 type 2 diabetes patients were included. Overall, 34% of participants had a poor understanding of hypoglycemia. Poor knowledge was linked to older age, illiteracy, and poor socioeconomic status [19]. According to a survey that included 2530 type 2 diabetes patients and was done by the American Association of Clinical Endocrinology, despite the fact that half or more of the research participants had previously experienced hypoglycemia, many patients had no idea of the causes or conditions [20]. Other research done in Najran showed that 44% of diabetics had limited knowledge of hypoglycemia symptoms [21].

Type 2 DM is now becoming a worldwide problem in healthcare. And the current findings show that improving diabetic patients' awareness, knowledge, and attitude toward the disease could result in improved glycemic control. It will help people to understand the risk of diabetes, motivate them to seek appropriate medical care, and teach them how to control the disease [22]. In the context of this, understanding the symptoms of hypoglycemia and being aware of potential preventative measures would be beneficial to type 2 diabetic care. We proposed to investigate type 2 diabetes patients' understanding of hypoglycemia in the Al Qassim region.

## Materials and methods

Study design

**Figure 1:**
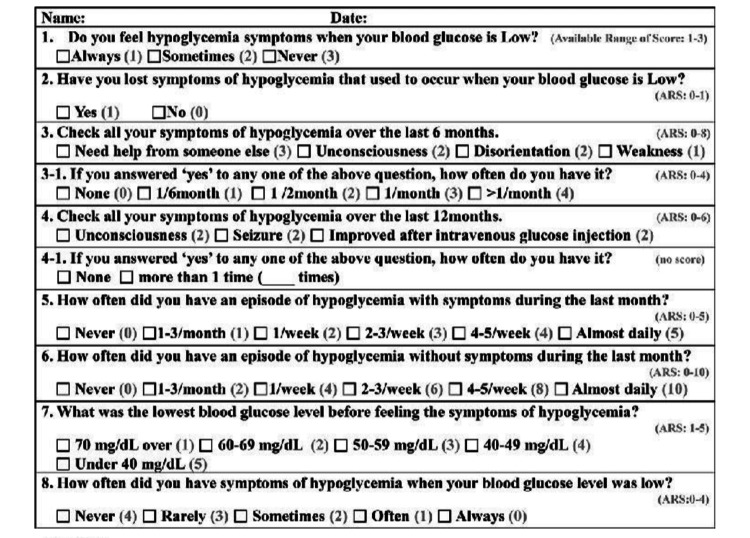
Modified Clarke's questionnaire

**Figure 2:**
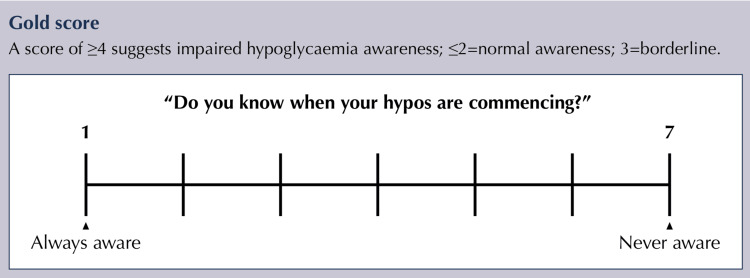
Gold questionnaire

This cross-sectional study was conducted in Al-Qassim, Kingdom of Saudi Arabia, from January to June 2022. We collected the data using a validated, self-administrator questionnaire and modified Clarke's and Gold questionnaires (Figure 1 and Figure 2). We conducted this through social media to assess knowledge and attitude toward hypoglycemic attacks.

Study setting

The data were collected through a previously published validated online questionnaire about knowledge and attitude toward hypoglycemic attacks.

Sample size

The sample size was calculated by using the EPI-Info app (Centers for Disease Control and Prevention, Atlanta, Georgia). The estimated sample size was 312. We used a simple random sampling technique by selecting patients from different areas in Al-Qassim. People who have diabetes mellitus type 2 and were on treatment with either oral hypoglycemic agents or insulin despite their gender and duration of disease were included, whereas people who were diagnosed with diabetes mellitus type 1 and gestational diabetes were excluded.

Data collection methods

We collected the data using a validated, self-administrator questionnaire. The questionnaire was translated into Arabic. We conducted it through social media. We targeted different cities in the Al-Qassim region to increase the chance of generalizing the findings. We obtained informed consent and assured that confidentiality was maintained on the first page of our questionnaire. The questionnaire is divided into three sections: the first section includes socio-demographic data (age, gender, nationality, the city where they live, education level, occupation, and marital status). The second section is Clarke's questionnaire, which comprises eight questions assessing awareness of hypoglycemia. The total score ranges from "0" to "7," and a higher score indicates impaired awareness. The four or more points indicate impaired awareness of hypoglycemia (IAH). The third section of the gold questionnaire is a single question, "Do you know when your hypos are commencing?" to which the participant responds on a seven-point Likert scale (1 = Always aware of hypoglycemia, 7 = Never aware of hypoglycemia). A score ≥4 suggests IAH.

Pilot study

A pilot study was conducted on 20 participants to estimate the clarity of data collection tools and the timing for data collection.

Data analysis plan

Data analysis was performed using SPSS (version 23; IBM Corp., Armonk, NY). The chi-square test was used for analyzing qualitative data. A p-value of 0.05 or less was considered statistically significant.

Study limitations

This questionnaire is self-administered by respondents, so it may be influenced by a recall bias.

Ethical considerations

Ethical approval was obtained from the Qassim Research Ethics Committee. We obtained informed consent and ensured that confidentiality was maintained.

## Results

Characteristics of the participants

**Figure 3:**
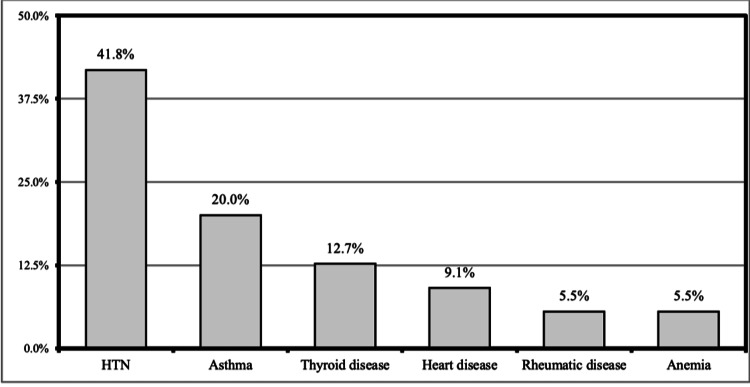
Frequency of chronic diseases among the study participants (n=55) HTN: hypertension

A total of 213 individuals participated in the study. They were predominantly female (70.9%). According to our findings, participants were 35.9 +/-13.0 years old on average. In addition, we saw that the majority of participants (73.7%) had university-level education. We discovered that roughly one-third of the study population was from Al Rass city when it came to geographic distribution. Then, 21.1% of them were from Unaizah city while 28.6% were from Buraidah city. Table 1 displays the distribution for additional cities. Additionally, our results showed that the majority of participants had no history of chronic illness (Table 1). Figure 3 shows the frequency of chronic diseases among the study participants.

**Table 1:** Socio-demographic characteristics of the participants (n=213)

Variable	Category	Frequency	Percent
Gender	Male	62	29.1%
	Female	151	70.9%
Educational level	Primary & illiterate	11	5.2%
	Intermediate	6	2.8%
	Secondary	35	16.4%
	Diploma	4	1.9%
	University	157	73.7%
City	Buraidah	61	28.6%
	Unaizah	45	21.1%
	Al Rass	68	31.9%
	Al Mithnab	3	1.4%
	Al Bukayriyah	5	2.3%
	Al Badayea	14	6.6%
	Asyah	1	0.5%
	Uyun Al Jiwa	1	0.5%
	Riyadh Al Khabra	10	4.7%
	Uglat Asugour	3	1.4%
	Dariyah	2	0.9%
History of chronic disease	Yes	55	25.8%
	No	158	74.2%

Awareness of hypoglycemia

According to our findings, the study population's average hypoglycemic awareness score, calculated using Clarke's questionnaire, was 3.6 +/-1.1 (Range 1-7). We used the Gold questionnaire, and the typical awareness score was 3.7 +/- 2.1. (Range 1-7). According to our research, the prevalence of impaired awareness of hypoglycemia (IAH) of study subjects who had a score of >/= 4 in the Clarke's and Gold questionnaires was 52.1% and 53.5%, respectively.

Our research revealed that most participants occasionally had hypoglycemia symptoms when their blood sugar level was low. The majority of individuals (62.4%) did not completely lose the hypoglycemia symptoms they used to experience when their blood glucose level was low. In addition, over half of the respondents noted weakness as a sign of hypoglycemia in the previous six months while 30.5% mentioned confusion, 10.3% mentioned unconsciousness, and 8% required assistance. In terms of the frequency of these symptoms, we discovered that the majority of respondents experienced them once every six months.

When we evaluated individuals' symptoms over the course of the previous year, we found that unconsciousness was reported by 46.9% of participants, improvement after an intravenous glucose injection by 31.9% of people, and seizures by 21.1% of participants. More than half of respondents acknowledged having these symptoms more than once. Our results showed that the majority of subjects had no episodes of hypoglycemia in the previous month, either with or without symptoms. Twenty-three percent (23%) of them had periods of hypoglycemia without symptoms while 26.8% of them had episodes of hypoglycemia lasting one to three months.

When we asked people what their lowest blood sugar level was before experiencing hypoglycemic symptoms, we discovered that 34.7% of them said it was above 70 mg/dl, which means they didn't have hypoglycemia and their symptoms were due to something else and 26.8% said it was between 50 and 59 mg/dl. Our results showed that just 6.6% of individuals experienced hypoglycemic symptoms when their blood glucose level was less than 70, and 36.6% of participants indicated that this happened occasionally and that they have hypoglycemia symptoms without any evidence of actual hypoglycemia (Table 2).

**Table 2:** Knowledge and awareness about hypoglycemia

Variable	Category	Frequency	Percent
1. Do you feel hypoglycemia symptoms when your blood glucose is low?	Always	56	26.3%
	Sometimes	128	60.1%
	Never	29	13.6%
2. Have you lost symptoms of hypoglycemia that used to occur when your blood glucose is low?	No	133	62.4%
	Yes	80	37.6%
3. Check all your symptoms of hypoglycemia over the last 6 months	Disorientation	65	30.5%
	Weakness	109	51.2%
	Unconsciousness	22	10.3%
	Need help from someone else	17	8%
3-1. If you answered “yes” to any one of the above questions, how often do you have it?	None	46	21.6%
	Once / 6 months	50	23.5%
	Once / 2 months	42	19.7%
	Once / month	41	19.2%
	> Once / month	34	16%
4. Check all your symptoms of hypoglycemia over the last 12 months	Unconsciousness	100	46.9%
	Seizure	45	21.1%
	Improved after I/V glucose injection	68	31.9%
4-1. If you answered “yes” to any one of the above questions, how often do you have it?	None	105	49.3%
	More than 1 time	108	50.7%
5. How often did you have an episode of hypoglycemia with symptoms during the last month?	Never	76	35.7%
	1-3 / month	57	26.8%
	1 / week	53	24.9%
	2-3 / week	17	8%
	4-5 / week	3	1.4%
	Almost daily	7	3.3%
6. How often did you have an episode of hypoglycemia without symptoms during the last month?	Never	91	42.7%
	1-3 / month	49	23%
	1 / week	42	19.7%
	2-3 / week	22	10.3%
	4-5 / week	5	2.3%
	Almost daily	4	1.9%
7. What was the lowest blood glucose level before feeling the symptoms of hypoglycemia?	> 70 mg/dl	74	34.7%
	60-69 mg/dl	43	20.2%
	50-59 mg/dl	57	26.8%
	40-49 mg/dl	17	8%
	< 40 mg/dl	22	10.3%
8. How often did you have symptoms of hypoglycemia when your blood glucose level was low?	Always	14	6.6%
	Often	20	9.4%
	Sometimes	78	36.6%
	Rarely	61	28.6%
	Never	40	18.8%

Factors associated with the prevalence of impaired awareness of hypoglycemia

Our findings showed that there was no correlation between age, gender, education levels, geographic distribution, or a history of chronic illness and the prevalence of decreased awareness of hypoglycemia. The prevalence of impaired awareness of hypoglycemia showed a relatively higher degree of association with geographic distribution and educational levels (P values = 0.088* and 0.187*, respectively) (Table 3).

**Table 3:** Factors associated with the prevalence of impaired awareness of hypoglycemia (IAH) * P values were calculated using Fisher’s exact test while other p values were calculated using the chi-square test.

Variable	Category	Prevalence of IAH			
		Gold Q n (%)	P value	Clarke Q n (%)	P value
Age (in years)	20 - 25	41 (60.3)	0.350	36 (52.9)	0.930
	26 - 35	17 (41.5)		19 (46.3)	
	36 - 45	31 (55.4)		30 (53.6)	
	46 - 55	18 (56.3)		18 (56.3)	
	> 55	7 (43.8)		8 (50)	
Gender	Male	33 (53.2)	0.956	37 (59.7)	0.157
	Female	81 (53.6)		74 (49)	
Educational level	Primary & illiterate	6 (54.5)	0.349*	5 (45.5)	0.187*
	Intermediate	4 (66.7)		3 (50)	
	Secondary	24 (68.6)		22 (62.9)	
	Diploma	2 (50)		0 (0)	
	University	78 (49.7)		81 (51.6)	
City	Buraidah	33 (54.1)	0.519*	25 (41)	0.088*
	Unaizah	19 (42.2)		29 (64.4)	
	Al Rass	38 (55.9)		39 (57.4)	
	Al Mithnab	2 (66.7)		2 (66.7)	
	Al Bukayriyah	4 (80)		4 (80)	
	Al Badayea	9 (64.3)		6 (42.9)	
	Asyah	1 (100)		1 (100)	
	Uyun Al Jiwa	0 (0)		0 (0)	
	Riyadh Al Khabra	6 (60)		2 (20)	
	Uglat Asugour	2 (66.7)		2 (66.7)	
	Dariyah	0 (0)		1 (50)	
History of chronic disease	Yes	30 (54.5)	0.860	25 (45.5)	0.251
	No	84 (53.2)		86 (54.4)	

## Discussion

The present study aimed to assess the knowledge and awareness about hypoglycemia as a complication of T2DM among the general population in the Al Qassim region, Saudi Arabia. It is difficult to achieve optimal blood glucose control since it involves the necessity for glycemic control with the risk of hypoglycemia [23,24]. Despite the increasing use of insulin to treat T2DM, hypoglycemia and IAH are not thought to pose substantial issues in the treatment of insulin-treated type 2 diabetes (T2DM). It has been proven that the duration of insulin treatment directly correlates with the risk of hypoglycemia in this population [25,26].

Our results revealed that the average awareness score of the study population was found to be 3.6 ± 1.1 (by using Clarke's method) and 3.7 ± 2.1 (by using the Gold method), which indicated that our respondents had inadequate knowledge of hypoglycemia. This was consistent with another study that was conducted in Saudi Arabia, which stated that participants (61.4%) had good knowledge of hypoglycemia, but only 38.6% had poor knowledge [27]. Another Indian study reported that 66.1% of diabetic patients had good knowledge of hypoglycemia [19]. Furthermore, we found that the prevalence of impaired awareness of hypoglycemia (IAH) by Clarke's questionnaire was 52.1% and 53.5% by using the Gold questionnaire. These results were lower than another study in Turkey, which showed that 83.4% had impaired awareness of their hypoglycemia [28], but higher than other studies in the UK (8%) [29], Brazil (35%) [30], and Malaysia (36.9%) [31]. These differences are most likely due to variations in scoring systems and the variability of the sampled population. Any hypoglycemia symptom could manifest, and typical symptoms aren't necessarily the first to show up [32]. Patients must therefore be aware of any signs in order to identify them early and take the necessary action. According to our research, weakness was the hypoglycemic symptom that people most frequently reported experiencing in the previous six months. Dizziness and weakness were the most prevalent signs of hypoglycemia among the study individuals, according to a previous Indian study [19]. In addition, we found that most respondents only encountered it once every six months.

In a prior study conducted in Brazil, more hypoglycemia episodes were discovered. During the four-week follow-up period, 61.8% of T2DM patients experienced at least one hypoglycemic incident [30]. Another study in the UK discovered that severe hypoglycemia had occurred in 15% of people the year before, with an estimated incidence of 0.28 episodes/patient/year for the general population [29]. In addition, we found that 23% experienced one to three episodes of asymptomatic hypoglycemia every month. In similar research, 10.6% of T2DM patients were found to have asymptomatic hypoglycemia [30]. The prevalence of poor awareness of hypoglycemia was found to have no significant association with age, gender, education levels, geographic distribution, or history of chronic illness. Another study, however, found that older age, illiteracy, and low socioeconomic position were related to poor knowledge, but insulin combined with orally administered antihyperglycemic agents (OHAs) was associated with strong knowledge [19]. This disparity may be attributable to differences in research population characteristics, as the majority of our study participants were young.

Our study had some limitations, including the potential for question misinterpretation, patients' inaccurate self-reporting of hypoglycemic events, and recall bias, particularly for baseline questions referring to the previous six months. Information is based on how well patients can recall and understand the signs and symptoms of low blood sugar.

## Conclusions

Our study revealed that type 2 diabetic patients in Saudi Arabia's Al-Qassim region lacked knowledge about hypoglycemia as a consequence of T2D. Furthermore, there was a high prevalence of impaired awareness of hypoglycemia. The current research highlights the value of patient education and doctor knowledge in the management of hypoglycemia, specifically the burden of hypoglycemic unawareness. To raise public awareness, interventional programs are required.
